# Wild Pedigree exploreR (wpeR): Streamlined Analysis and Visualization of Wild Pedigrees in Time and Space

**DOI:** 10.1111/1755-0998.70171

**Published:** 2026-06-23

**Authors:** Gregor Simčič, Tomaž Skrbinšek

**Affiliations:** ^1^ Biotechnical Faculty University of Ljubljana Ljubljana Slovenia; ^2^ DivjaLabs Ltd. Ljubljana Slovenia

**Keywords:** non‐invasive genetic sampling, pedigree reconstruction, pedigree visualization, population ecology, R package, wildlife genetics

## Abstract

Advances in non‐invasive genetic sampling and long‐term genetic monitoring programmes have enabled collection of large individual genotype datasets for many wildlife populations, often accompanied by rich field metadata that place the genotyped individuals in time and space. These datasets allow reconstruction of multigenerational pedigrees and have the potential to provide valuable insights into population demography, reproduction, dispersal, social structure and genetic processes. But while the tools for construction of pedigrees keep improving, their interpretation remains challenging. Integrating multigenerational pedigree data with field metadata creates significant complexity, yet specialized tools to facilitate the interpretation of such datasets remain scarce. Here we introduce wild pedigree exploreR (wpeR), an R package designed to simplify exploration, organization and interpretation of complex pedigrees. The package enables users to link reconstructed pedigrees with genetic sample metadata, enabling evaluation of biological plausibility of inferred relationships, but also allowing exploration of other characteristics of individuals and populations in spatial and temporal contexts. wpeR implements a linear workflow through which the pedigree data is imported, formatted, organized into families and integrated with field metadata. The resulting dataset can be visualized through temporal plots that track individuals and families over time, as well as with spatial outputs representing parent–offspring relationships and individual movement patterns as geographic features that can be either directly visualized on maps within R, or exported to be further explored with common GIS tools. wpeR allows exploration of lineage relationships within their ecological context, bridging the gap between statistically reconstructed pedigrees and their biological interpretation. It provides a scalable and flexible framework for analyzing these complex data, providing a practical tool for researchers and managers working with genetic monitoring datasets.

## 1 Introduction

The advent of genetic data in wildlife research has significantly advanced our ability to understand the processes within animal populations. Particularly, the development of methods that enable extraction of genetic data from non‐invasive samples has opened new opportunities for studying a wide array of vertebrate species, including those that are rare and elusive (Piggott and Taylor 2003; Beja‐Pereira et al. 2009). The technical and scientific advances that enabled this genetic revolution have led to the development of sophisticated genetic monitoring programmes and research projects (Schwartz et al. 2007; Carroll et al. 2018; Zemanova 2021). Such efforts have often managed the genotyping of a substantial proportion of specific animal populations, resulting in the accumulation of extensive individual genotype data across multiple generations. This wealth of data, coupled with the emergence of novel statistical approaches, has enabled researchers to reconstruct complex pedigrees with considerable accuracy and depth (Pemberton 2008; Jones et al. 2010; Flanagan and Jones 2019).

By combining non‐invasive genetic sampling methods, long‐term wildlife monitoring schemes and the reconstruction of pedigrees, researchers now have the ability to study key ecological, behavioral and evolutionary characteristics across a broad spectrum of species with just one type of data. This approach enables detailed studies of demography (De Barba et al. 2010; Caniglia et al. 2014), reproductive dynamics (Carpenter et al. 2005; Constable et al. 2001), dispersal patterns (Jarausch et al. 2021; DeMay et al. 2017), inbreeding, hybridization and other population genetic parameters (Liberg et al. 2005; Quinn et al. 2019). Furthermore, the integration of these techniques can also help us gather insights into animal social systems (Stanton et al. 2015; Granroth‐Wilding et al. 2017). Such data can also serve as a resource for connectivity studies (Kormann et al. 2012) and forms a foundation for setting specific genetic management goals and strategies (McLennan et al. 2018).

Although the current genetic toolkit elevated ecological research to a new level, it does not represent a panacea for the study of animal populations. As in other fields of ecology, the development of new methods and research techniques gave rise to new challenges, many of which are connected with parsing and editing large amounts of data (Farley et al. 2018). In the framework of ecological research, the reconstruction of pedigrees typically signifies one of the last phases of a complex workflow that includes study design, field sampling, laboratory processing and identification of individuals (Jones et al. 2010). However, the reconstructed pedigree is not the end, but rather a pivot to a new phase of investigation. It marks the beginning of an analysis phase, where researchers engage in deciphering patterns driving animal population dynamics or finding answers to other questions motivating the research in the first place.

Regardless of the selected method or software for pedigree reconstruction (for reviews of methods and software see Jones et al. 2010; Flanagan and Jones 2019), the analytical phase faces several challenges, the foremost being the assessment of pedigree accuracy (Pemberton 2008). An essential step in this process is the understanding and consideration of the underlying assumptions of the chosen pedigree reconstruction method (Koch et al. 2008; Jones and Wang 2010b; Walling et al. 2010). Additionally, researchers must pay attention to the estimation of genotyping error rates and the proportion of sampled candidate parents (Jones et al. 2010). Prior to finalizing the pedigree, these factors should be evaluated. However, the task of constructing pedigrees from a dataset that also includes data from noninvasive genetic samples often presents unique challenges (Wang 2019). Frequently, the assumptions underlying standard reconstruction methods may not fully apply to such data sets, which necessitates further scrutiny. In such cases, it becomes imperative to conduct a thorough examination of the spatial and temporal coherence of the pedigree. This examination not only ensures the accuracy and relevance of the pedigree but can also offer insights into behavioral patterns as well as reproductive, social and spatial dynamics of the population under study. However, to gain insights from pedigrees obtained from wild populations, we need to understand them within their spatial and temporal dimensions. Without specialized software tools, this is a very daunting task.

To achieve these insights, a pedigree needs to be connected with genetic sample metadata that stores the information on the sample collection date, location and other parameters recorded at the time of collection of a sample. This dataset can then be visualized using spatial and temporal visualizations that allow us to interpret complex lineage relationships and interactions, uncovering patterns in population dynamics and behavioral ecology.

This is what our package is designed to do. Wild pedigree exploreR—wpeR—is an R (R Core Team 2025) software package designed to deal with the complexities posed by the analysis and interpretations of multigenerational pedigrees of wild animals.

wpeR facilitates a streamlined and efficient analysis workflow for researchers dealing with complex wild pedigree data. It adds spatial and temporal dimensions to the pedigree, providing the researcher with detailed insights into dynamics and development of the population by tracking both lineage and movement of each detected individual in space and time. The package also offers a standardized procedure for analyzing such data and provides outputs that are in formats which can be further analyzed or visualized with existing R packages or other software.

The wpeR workflow is designed to complement the statistical assessment of the pedigree accuracy. Its strength lies in streamlining essential steps: the assessment of biological and spatiotemporal plausibility of the pedigree, and its biological interpretation. By integrating reconstructed pedigree with field metadata, wpeR allows researchers to use their knowledge about the species and population they are studying, and specifics of their study, to visually examine and validate inferred relationships within their real‐world context, bridging the gap between statistical output and biological reality.

## 2 Materials and Methods

### 2.1 Overview of the Workflow

**FIGURE 1:**
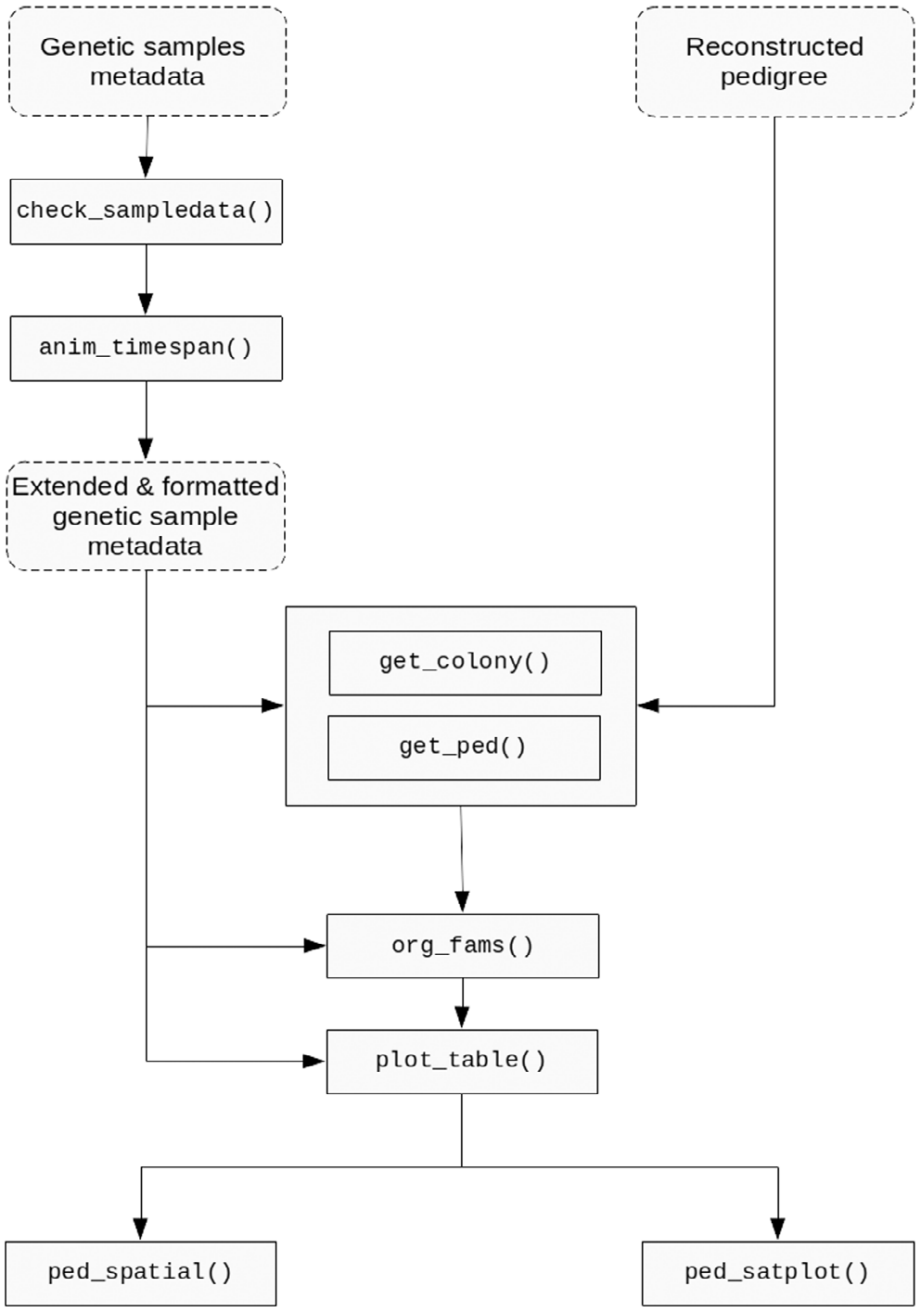
Main functions of the wpeR package and the example of the usage workflow. Full line rectangles represent functions, dotted line rectangles represent input datasets. Arrows represent which function outputs serve as input in subsequent functions.

Much of the functionality of the package is designed to follow a linear progression, where the output of one function serves as the input for the subsequent one (Figure 1). This sequential approach ensures a coherent and streamlined analysis of the data while keeping individual steps independent. Importantly, the intermediate results derived from these functions are not confined exclusively to the internal processes of the package. Rather, they are intentionally designed to be exportable, offering users the flexibility for further analysis using additional R packages or other software. This design choice acknowledges the diverse analytical needs of researchers and encourages exploration of additional methods outside the package framework.

### 2.2 Input Data

The two main inputs required to use wpeR functions are: (1) reconstructed pedigree and (2) genetic samples metadata (and/or other individual‐specific data with spatial and temporal information). Pedigree can either be an output of Colony (Jones and Wang 2010a) pedigree reconstruction software or a custom data frame. Custom pedigree must include three columns named OffspringID, FatherID and MotherID, with unknown parents represented by NA values. Genetic samples metadata must include the minimum required information on all genetic samples that belong to the individuals included in the pedigree, where an individual can be sampled multiple times (which is usually the case in data obtained from genetic monitoring programmes). The package requires that this information includes identifier codes for samples and individuals, sex of individual, sample collection date, geographic location of the sample, type of the sample and a logical column that marks samples collected from dead animals (Table 1). Besides the above mentioned information, other attributes related to the genetic samples can be included, but those are not crucial for the functioning of the package. Other data (e.g., telemetry data, in some cases photo trap data) can also be added as long as it is connected to the genetically identified individual in the pedigree. While geographic coordinates are currently a requirement for spatial mapping and downstream processing, users working with non‐spatial datasets can still utilize the package's tabular sorting and non‐spatial visualization features. This can be achieved by providing general or placeholder coordinates in the input metadata (e.g., the centroid of the study area or dummy values).

**TABLE 1:** Essential metadata for genetic samples when using wpeR package.

Data column	Explanation
Sample unique identifier code	
Date of sample collection	YYYY‐MM‐DD format
Individual identifier code	
Genetic sex	M for males, F for females and NA for unknown sex
Latitude	WGS 84 coordinate system
Longitude	WGS 84 coordinate system
Sample type	eg.: scat, urine, tissue
Mortality sample	TRUE or FALSE

Precise formatting of metadata is essential for ensuring accurate functionality of the package as the procedures applied by the package functions critically depend on the correct formatting. To help with this requirement, the package includes a dedicated function, check_sampledata(), which checks if all the needed information is included and outputs a correctly formatted data frame.

After fulfilling the data format requirements, all individuals can be assigned detection timespan and mortality identification (time period when they were detected and whether mortality was detected) with the anim_timespan() function. This information is needed in downstream functions.

### 2.3 Tabular Outputs

To import the pedigree, the user has two options. If the pedigree was reconstructed using the Colony software, the get_colony() function should be used. This function extracts the necessary data directly from the Colony project path. In cases where the pedigree was reconstructed using other software, the user must initially format it to align with the basic pedigree structure that has three columns corresponding to information about offspring, father and mother individuals. Subsequently, the user can import it using the get_ped() function. Both functions enable the user to transform the pedigree into formats needed for subsequent analysis and visualization with kinship2 (Sinnwell et al. 2014), pedtools (Vigeland 2021) and FamAgg (Rainer et al. 2016) R packages.

The next step in the package workflow involves grouping individuals into families and groups of paternal and maternal sibs and half‐sibs. This step introduces two foundational concepts used throughout the package: ‘family’ and ‘half‐sib group’. A ‘family’ is defined as a group of animals where at least one parent and at least one offspring are known. Meanwhile, a ‘half‐sib group’ refers to a group of half‐siblings who are either maternally or paternally related, but where we know that they differ in one of the parents. The grouping process is executed through the org_fams() function, producing two outputs. The first output named fams is a data frame indexing all identified families within the provided pedigree, accompanied by supplementary details. These details include information on reproductive individuals of the family, the initial detection date of any offspring, the latest detection date of either reproductive male or female of that family, and whether the family can be considered no longer reproducing because of the recorded death of any of the parents. Additionally, half‐sib relationships are outlined through paternal or maternal relatedness of the families. The second output of the org_fams() function is an extended pedigree named ped. Within the ped data frame, each individual included in the pedigree is assigned attributes from the fams index of families. The result is a more comprehensive and informative pedigree, where each individual's affiliations, familial history and associated attributes contribute to the understanding of the population structure and dynamics.

Building upon the family and individual attributes integrated into the pedigree, the next step in the workflow involves preparing the data for the temporal and spatial representation. This is done with the plot_table() function. This function automates the data preparation step for acquiring temporal and spatial insights through visualization. The function takes all the attributes of the extended pedigree and joins them with the field metadata (genetic samples metadata, telemetry locations, etc.). By merging the extended pedigree with sample metadata, the information from the pedigree becomes linked with the dates and locations of the samples.

To maintain the interpretability of complex pedigrees, the plot_table() function includes two filtering options for selective visualization. Users can utilize the plot_fams argument to focus on specific families or use the plot_indivs argument to isolate all families associated with specific individuals. For example, by passing a parent's identifier to plot_indivs, the function recovers their entire reproductive history, including all maternal or paternal half‐sib groups (identified also via the MomHSgroup or DadHSgroup attributes in the output of the org_fams() function). This functionality allows researchers to separate complex relationships into manageable lineages, preventing visual clutter in the final temporal (ped_satplot()) and spatial (ped_spatial()) representations.

### 2.4 Visualizations

After the tabular data preparation stage, the data can be visualized. wpeR offers two options for visualizing the dynamics within a pedigree dataset: a temporal representation using the ped_satplot() function, or a spatial representation which can be obtained through the ped_spatial() function.

#### 2.4.1 Temporal Pedigree Plot

**FIGURE 2:**
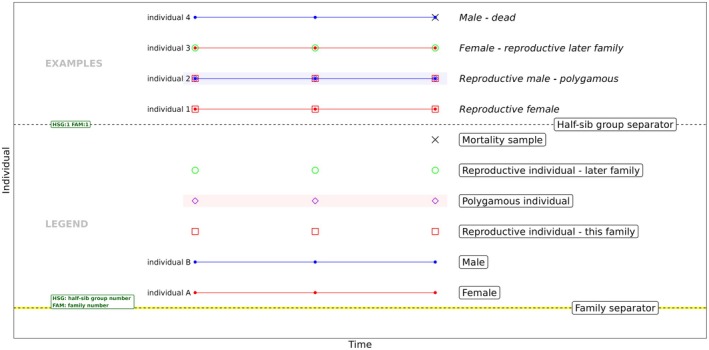
Example temporal plot produced by wpeR package. Each line is an individual, and each symbol on that line is a single detection of that animal (genetic sample). Time moves from left to right. The top half illustrates a basic example of plotted individuals; the bottom half serves as a legend, explaining various symbols and markings used in the plot.

The core of the temporal plot (Figure 2), generated by the ped_satplot() function, is the representation of the occurrence of samples for each individual (*y*‐axis) through time (*x*‐axis). The individuals are first grouped by families and then by half‐sib groups. Within each family, the individuals are arranged from top to bottom based on the date of their first sample collection. Positioned at the bottom of each family plot is the individual that was detected first, followed by subsequent individuals in chronological order. Such a layout enables a visual understanding of the temporal relationships within and between families, with each family forming a distinct cluster in the plot.

Each sample (detection) is visually depicted as a point on the plot, and points (samples) of the same individual are connected by lines to represent the survival/tracking history of that individual. Each sample is additionally marked to represent any characteristics of a particular individual (e.g., reproductive animal, polygamous animal—animal known to have offspring with several mates). User‐predefined mortality samples (where mortality was detected) are marked to indicate mortality. Under the hood, the ped_satplot() function harnesses the capabilities of ggplot2 (Wickham 2011) for the creation and rendering of the temporal plot, making it easy for the user to enhance or customize the plot using the familiar ggplot2 syntax.

#### 2.4.2 Spatial Data

The ped_spatial() function incorporates the spatial dimension into the pedigree analysis. Acting as a wrapper function, ped_spatial() combines multiple functions that utilize the output of the plot_table() function, transforming it into various sf (simple features) objects (Pebesma 2018) that can be visualized on a map. Using the default function parameters, the ped_spatial() function produces a list containing 14 sf objects (Table 2). Through the integration of POINT, LINESTRING and POLYGON geometries, the function generates sf objects that establish connections between parent and offspring samples, as well as the temporal connections between samples of the same individual.

**TABLE 2:** List of the 14 sf objects produced by the ped_spatial() function. The name of the object is composed from the role and data type labels (e.g., MotherRpoins).

Object name		Description
Role	Data type	
Mother	Rpoints	POINT object representing reference samples of each animal. Reference sample for parents is their chronologically last sample, reference sample for the offspring is their first sample within the user defined time frame
Father		
Offspring		
Mother	MovePoints	POINT object representing all samples of a particular animal
Father		
Offspring		
Maternity	Lines	LINESTRING object connecting reference samples of mothers or fathers with reference samples of their offspring
Paternity		
Mother	MoveLines	LINESTRING object connecting all samples of an individual in chronological order, showcasing the movement or changes in location over time for the specific animal
Father		
Offspring		
Mother	MovePolygon	POLYGON object representing a convex hull that encloses all the samples of an individual. It provides a graphical representation of the spatial extent or range covered by the animal based on its sample locations
Father		
Offspring		

When generating spatial files, the user can limit the timeframe from which the samples are included in the spatial representation. By adding this temporal constraint, the package allows one to focus the analysis on specific periods of interest.

The default output of the ped_spatial() function is a list of sf objects that can be further analysed and visualized using R packages that enable visual representation of geographic datasets such as leaflet (Cheng et al. 2023) and mapview (Appelhans et al. 2023). To extend the possibilities of spatial analysis and visualization outside of R, ped_spatial() provides an option to directly export the spatial data in file formats compatible with geographic information system (GIS) software.

## 3 Results

To demonstrate the practical application of the package, we provide a usage example from a real‐world ecological dataset that is included with the package. This demo dataset included in the package is taken from the data obtained through the Slovenian national grey wolf ( *Canis lupus* ) monitoring programme. It consists of genetic samples metadata (wolf_samples) and a pedigree reconstructed with Colony software (wpeR_samplePed).

The genetic samples metadata within this dataset includes 278 samples from 65 individuals, collected between 2019 and 2021. The included dataset is a small subset of the complete dataset of the wolf monitoring programme. This subset was intentionally produced to serve as a demonstration example, showcasing as many functionalities of the wpeR package as possible while using a minimal amount of data to simplify the analysis.

The example follows the basic workflow outlined in the previous chapter. (1) Get animal timespans and mark dead animals. (2) Merge the timespan data with genetic sample metadata. (3) Import/format the pedigree. (4) Organize families and expand the pedigree. (5) Join the extended pedigree attributes with genetic samples metadata and prepare data for visual representation. (6) Generate temporal plots and/or spatial files.># (1)Get animal timespan data using the `anim_timespan()` function.>animal_ts <- anim_timespan(   individual_id = wolf_samples$AnimalRef,   sample_date = wolf_samples$Date,   mortality_sample = wolf_samples$IsMortality )># (2) Add animal timespan to the sampledata >sampledata <- merge(   wolf_samples,   animal_ts,   by.x = "AnimalRef",   by.y = "ID",   all.x = TRUE )># (3) Retrieve the pedigree data from the get_colony function.>path <- paste0(system.file("extdata", package = "wpeR"), "/wpeR_samplePed")>ped_colony <- get_colony(   colony_project_path = path,   sampledata =  wolf_samples,   rm_obsolete_parents = TRUE,   out = "FamAgg" )># (4) Organize families and expand pedigree data using the org_fams function.>ped_org <- org_fams(   ped = ped_colony,   sampledata = sampledata,   output = "both" )># (5) Prepare data for plotting.>pt <- plot_table(   plot_fams = 1,   all_fams = ped_org$fams    ped = ped_org$ped,   sampledata = sampledata)


The plot_table() function provides two primary arguments for subsetting data: plot_fams for isolating specific family units, and plot_indivs to automatically extract families associated with specific individuals. Because the output of plot_table() serves as the direct input for both ped_satplot() and ped_spatial(), these arguments allow users to control the complexity of the final visual representation. While there is no technical limit to the number of families that can be processed simultaneously, filtering is crucial to balancing the number of represented families and visual interpretability. As pedigree complexity increases, graphs can easily become oversaturated. This built‐in filtering flexibility allows researchers to bypass visual clutter by seamlessly breaking down complex pedigrees into manageable groups, visualizing maternal or paternal histories separately or exploring the dataset one family at a time.

**FIGURE 3:**
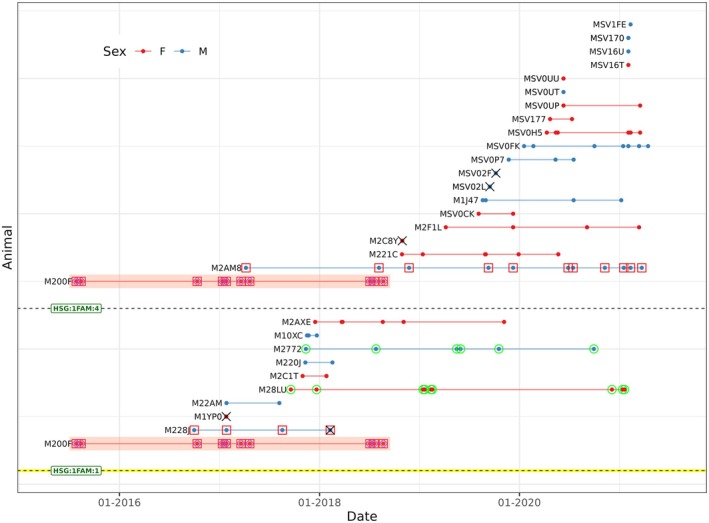
wpeR temporal plot showing two wolf packs that share the same mother. Each individual is in it's horizontal line, each symbol is a detection (sample). Blue = males, red = females. For the detailed explanation of the symbols see Figure 2 and section Temporal pedigree plot.


Calling the ped_satplot() function, using the output of plot_table() as the sole input parameter provides the most straightforward method for generating a spatial plot. The result (Figure 3) is a ggplot2 object that can be further styled using the ggplot2 syntax.

># (6.1) Generate temporal plot.>sp <- ped_satplot(pt)



The example temporal plot in Figure 3 displays two families (FAM: 1 and FAM: 4) that are maternal half‐sibs. Family 1 is comprised of 10 individuals: the two parents (samples marked with red squares) and eight offspring (four males, four females). Family 4 consists of the parents and 18 offspring (nine males, nine females). The plot provides several biological insights. For instance, individuals marked with a black 5‘x’ are confirmed dead, which includes the reproductive male of Family 1. The temporal arrangement of samples illustrates that the reproductive female from Family 1 got a new mate after the death of the reproductive male from Family 1. Because this female reproduced with more than one male, she is classified as polygamous and her samples are marked with purple diamonds and red background. Furthermore, the green circles on two offspring individuals from Family 1 indicate that they have become reproductive in other families within the pedigree.

The exploration of the spatial dimension of the pedigree is facilitated by the ped_spatial() function. Like ped_satplot(), it accepts the output of plot_table() as its primary input. However, it is good practice to use additional arguments given the complexity of the functions' output.># (6.2) Generate spatial objects.>ps <- ped_spatial(   plottable = pt,   time.limits = c(as.Date("2017‐01‐01"), as.Date("2018‐01‐01")),   time.limit.rep = TRUE,    time.limit.offspring = TRUE,   time.limit.moves = TRUE )


**FIGURE 4:**
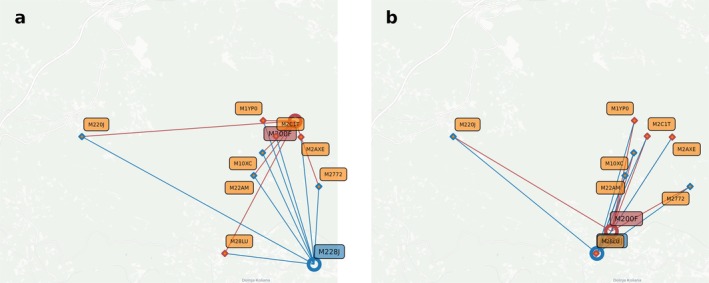
Visual representation of the first component of the ped_spatial() function, showing mother reference point (red hollow circle with the individual id shown as red rectangle), father reference point (blue hollow circle with the individual id shown as blue rectangle), offspring reference point (diamond with orange fill and red or blue outline, depending on the sex of the individual, the individual id is shown in orange rectangle), maternity lines (red) and paternity lines (blue). (a) shows all reference samples without time limits, (b) shows just samples that fall between the defined time limits (from 2017‐01‐01 to 2018‐01‐01).

The default output of the ped_spatial() function is a list containing 14 sf (simple feature) objects (Table 2). This format unlocks the full potential of the simple feature access standard (Open Geospatial Consortium 2011), offering versatility in spatial operations and enabling diverse visual representations. In the provided example of the function's output, we categorized them into three distinct components. The first component shows mother, father and offspring reference points along with maternity and paternity lines (Figure 4). This representation shows parental connections in space, allowing the user to visually trace the spatial relationships in the pedigree, discern the geographical proximity and understand the distribution of parental pairs and their respective offspring. This visualization is particularly advantageous for distinguishing between families that exhibit spatial and temporal overlap.

**FIGURE 5:**
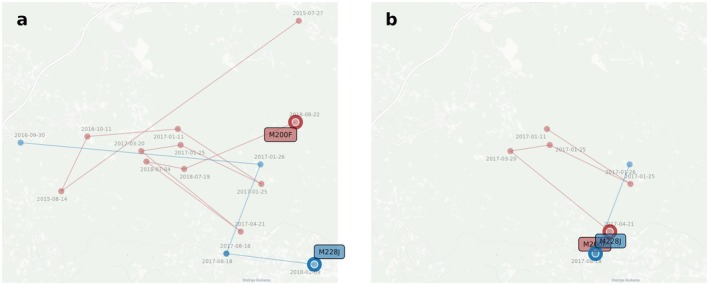
Visual representation of the second component of the ped_spatial() function output, showing mother reference point (red hollow circle with the individual id shown as red rectangle), father reference point (red hollow circle with the individual id shown as red rectangle), mother movement points (full red circles) and lines (red) as well as father movement points (full blue circles) and lines (blue). Each movement point is annotated with a sample collection date. Figure 4a shows all reference samples without time limits, Figure 4b shows just samples that fall between the defined time limits (from 2017‐01‐01 to 2018‐01‐01).

**FIGURE 6:**
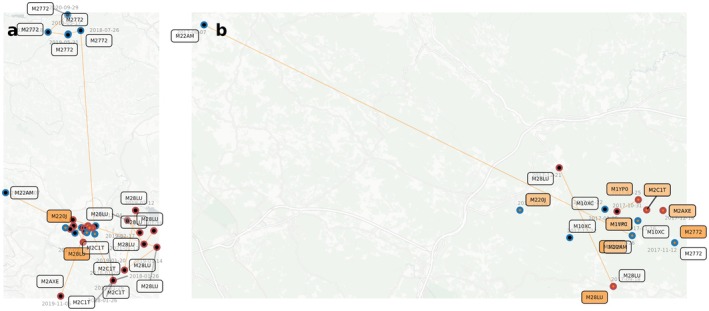
Visual representation of the third component of the ped_spatial() function output, showing offspring reference point (diamond with orange fill and red or blue outline, depending on the sex of the individual, the individual id is shown in orange rectangle), offspring movement points (circles with black fill and red or blue outline, depending on the sex of the individual) and offspring movement lines (orange). Each movement point is annotated with a sample collection date. Figure 4a shows all reference samples without time limits, Figure 4b shows just samples that fall between the defined time limits (from 2017‐01‐01 to 2018‐01‐01).

The second component shows mother and father reference points, their movement points as well as their movement lines, thus connecting all samples of both parents in chronological order (Figure 5). Similarly, the third component extends this representation to encompass all offspring (Figure 6). Through such movement representation, we can obtain a rough approximation of individual home ranges, particularly in the case of social/pack animals like wolves, providing insights into the rough extent of their territories. This approach allows us to track individual movements over time, gaining valuable insights into animal dispersion processes.

While ped_spatial() automates the task of joining pedigree attributes with geographic data, the resulting output is a standard list of sf objects, providing the user with full flexibility for visualization via ggplot2. A straightforward example of plotting a family's spatial history is shown below.>ggplot() +  geom_sf(data = ps$motherMoveLines, color = "#b7484b", linewidth = 0.5) +  geom_sf(data = ps$motherRpoints, color = "#b7484b", size = 4, shape = 1) +  geom_sf(data = ps$motherMovePoints, color = "#b7484b", size = 2) +  geom_sf(data = ps$fatherMoveLines, color =  "#1f78b4",  linewidth = 0.5) +  geom_sf(data = ps$fatherRpoints, color = "#1f78b4", size = 4, shape = 1) +  geom_sf(data = ps$fatherMovePoints, color = "#1f78b4", size = 2) +  geom_sf_text(data = ps$fatherMovePoints,    aes(label = Date), vjust = -0.8, size = 2) +  geom_sf_text(data = ps$motherMovePoints,    aes(label = Date), vjust = -0.8, size = 2) +  geom_sf_label(data = ps$motherRpoints,    aes(label = AnimalRef), size = 3, vjust = 1.5) +  geom_sf_label(data = ps$fatherRpoints,    aes(label = AnimalRef), size = 3, vjust = 1.5) +  theme_minimal()


While the basic example provides a quick visualization, production‐quality maps often require additional parameters for extent calculation and aesthetic refinement, or final map production using GIS software. For advanced static visualizations, environmental background layers can be integrated via the basemaps package (Schwalb‐Willmann 2024), while the mapview package can be utilized for dynamic, interactive overviews within the R environment. Detailed scripts for high‐quality map generation, such as those shown in Figures 4, 5, 6, are provided in the package vignette.

When observing all three components simultaneously in an interactive GIS environment, across an area with multi‐generational data, one can study population dynamics in spatial terms. This view enables the identification of sinks or sources of population growth or decline. Additionally, it allows for insights into the origins of animals establishing new home ranges, providing information about the contributing families and their roles in shaping the spatial structure of the population.

## 4 Discussion

The wpeR package provides a novel framework for the exploration and interpretation of complex wild pedigree data. Implemented as an R package and supported with worked examples in the vignette, it is simple and intuitive for researchers familiar with R to pick up and use in their own analyses. It is equally simple to extend its spatial visualization and analysis functionality using a GIS. The package provides an easily scalable solution for analysing pedigrees, from single‐family or one‐generation data to complex multigenerational pedigrees, with complete workflow executing in approximately 10 s for datasets of 400 individuals and 2000 samples on standard desktop hardware. The implementation of the wpeR workflow offers a step towards mitigating data variety challenges (Farley et al. 2018) connected with research and monitoring programmes where non‐invasive genetic sampling and pedigree reconstruction is used.

But as with any software, its performance will depend on input data quality and completeness. Any missing values in the required metadata attributes in Table 1 will prevent the package functions from executing correctly. All outputs will of course reflect the sampling effort, with more comprehensive and systematic sampling resulting in more complete and reliable insights. wpeR is mainly a tool for organization and visualization of wild pedigree data, and the interpretation of the outputs will be subjective. When used to check the pedigrees, the final judgment of biological plausibility of the outputs rests on the researcher and their expert knowledge of both the studied species' biology and the particulars of their study. In this regard, wpeR should be seen as complementary to other methods used for assessing the accuracy of a reconstructed pedigree.

Apart from these considerations, wpeR offers a new set of tools for researchers in the field of wildlife genetics and ecology. It allows them to increase their confidence in pedigrees reconstructed by various available methods by allowing them to visually inspect the spatiotemporal plausibility of the inferred relationships (Blouin 2003; Jones et al. 2010), offering a quality control step that helps prevent erroneous conclusions in downstream analysis. But beyond that, wpeR also offers new possibilities to visualize and interpret such data, providing opportunities to gain new biological insights. Efficiently visualizing these complex data can help reveal hidden patterns in dispersal, social structure, reproduction and other population parameters that could be missed in tables of data alone.

As far as we know, wpeR is the first tool with this functionality. As genetic monitoring increasingly becomes routine for many species and populations of conservation or management concern, multi‐generational data will continue to accumulate, becoming more and more valuable. We believe that wpeR will find its use with many such datasets, providing important insights for numerous species and circumstances.

## Author Contributions

T.S. conceived and designed the software. G.S. developed the package architecture, wrote the documentation, tests and prepared the usage example. Both authors contributed to writing the manuscript and approved the final version.

## Funding

This work was supported by European Commission. Slovenian Research and Innovation Agency P1‐0184.

## Conflicts of Interest

The authors declare no conflicts of interest.

## Data Availability

The data that support the findings of this study are openly available in CRAN at https://CRAN.R‐project.org/package=wpeR.
